# Genetic variations associated with response to dutasteride in the treatment of male subjects with androgenetic alopecia

**DOI:** 10.1371/journal.pone.0222533

**Published:** 2019-09-16

**Authors:** Arang Rhie, Ho-Young Son, Soo Jung Kwak, Seungbok Lee, Dong Young Kim, Bark-Lynn Lew, Woo-Young Sim, Jeong-Sun Seo, Ohsang Kwon, Jong-Il Kim, Seong Jin Jo

**Affiliations:** 1 Genomic Medicine Institute (GMI), Medical Research Center, Seoul National University, Seoul, Korea; 2 Genome Informatics Section, Computational and Statistical Genomics Branch, National Human Genome Research Institute, National Institutes of Health, Bethesda, Maryland, United States of America; 3 Department of Biomedical Sciences, Seoul National University College of Medicine, Seoul, Korea; 4 Department of Biochemistry and Molecular Biology, Seoul National University College of Medicine, Seoul, Korea; 5 Department of Dermatology, Seoul National University College of Medicine, Seoul, Korea; 6 Laboratory of Cutaneous Aging and Hair Research, Biomedical Research Institute, Seoul National University Hospital, Seoul, Korea; 7 Institute of Human-Environmental Interface Biology, Medical Research Center, Seoul National University College of Medicine, Seoul, Korea; 8 Department of Dermatology, College of Medicine, Kyung Hee University, Seoul, Republic of Korea; University of Minnesota, UNITED STATES

## Abstract

Dutasteride, a dual inhibitor of both type I and II 5α-reductases, is used to treat male pattern hair loss (MPHL). However, patient response to dutasteride varies in each individual, the cause of which is yet to be identified. To identify genetic variants associated with response to dutasteride treatment for MPHL, a total of 42 men with moderate MPHL who had been treated with dutasteride for 6 months were genotyped and analysed by quantitative linear regression, case-control association tests, and Fisher’s exact test. The synonymous single nucleotide polymorphism (SNP) rs72623193 in *DHRS9* was most significantly associated with response to dutasteride, followed by the non-synonymous SNP rs2241057 in *CYP26B1*. Additionally, variants in *ESR1*, *SRD5A1*, *CYP19A1*, and *RXRG* are suggested to be associated with response to dutasteride. Cumulative effect and interaction among these SNPs were presented in both additive and non-additive models.

## Introduction

Male pattern hair loss (MPHL) is an androgen-dependent progressive hair loss caused by shortening of anagen duration leading to miniaturization of involved hair follicles [1, 2]. About 80% of Caucasian men have MPHL by 70 years of age [3]. MPHL develops in genetically predisposed individuals. In a twin study, the heritability was estimated to be over 80% [4]. A polymorphism in the androgen receptor (AR) gene is associated with MPHL [5–10]. Recently, genome-wide association studies identified several loci associated with MPHL [9–13]. However, MPHL genetics are still not fully understood.

Androgen is the most important factor in the pathophysiology of MPHL. Although the major circulating androgen is testosterone, the androgen responsible for MPHL is dihydrotestosterone (DHT), which is synthesized from testosterone by 5α-reductase (5AR) in hair follicles and sebaceous glands. Thus, 5AR inhibition is a primary treatment target for MPHL. Varying amounts of hair regrow in MPHL patients treated with finasteride, a selective type II 5AR [14], and dutasteride, a dual inhibitor of both type I and type II 5ARs [15, 16],

However, 5AR inhibition does not always guarantee hair regrowth in the patients with MPHL, and response to medication varies in each individual. In a previous study, about 30% of patients treated with dutasteride for 6 months revealed ‘no global change’ as determined by investigator photographic assessment and subject self-assessment [15, 16]. The cause of the individual difference in response to 5AR inhibition for MPHL has not yet been identified.

In this study, we classified MPHL patients treated with dutasteride for 6 months into good- and poor-response groups according to the degree of hair regrowth and performed association analysis by targeting 154 genes related to the androgen pathway and drug metabolism.

## Materials and methods

### Subjects

This study protocol was approved by the Institutional Review Board of Seoul National University Hospital (H-1007-015-322). The target subjects were men with moderate MPHL (IIIv, IV, and V according to the modified Norwood-Hamilton classification scale) who had participated in the previous phase III study to evaluate the efficacy and safety of dutasteride (0.5 mg) [15]. They were treated with dutasteride or a placebo for 6 months. We contacted the 64 subjects who had been assigned to the dutasteride treatment group and had completed the clinical trial, and took peripheral blood samples from them after obtained a written informed consent. Their epidemiological characteristics and hair counts before and after treatment were obtained from the phase III study of dutasteride. To evaluate treatment response, the hair counts were measured at the same circular area marked with ink tatoo before and after 6 months of treatment (S1 Fig); that is, a substantial increase in hair count was considered a good response to dutasteride.

### Library construction and targeted sequencing

DNA was extracted from peripheral blood samples of the subjects. Target genes were carefully selected among genes known to be associated with MPHL in previous genetic studies including genome-wide association study and genes related to the steroid hormone biosynthesis (S1 Table) [5–10, 17–19]. In total, 154 genes were targeted (120 for whole gene and 34 for exon only) with the NimbleGen SeqCap EZ Choice Library. We performed sequencing with HiSeq2000 (Illumina, Inc. San Diego, CA, USA).

### Variant calling and SNP filtering

We aligned the DNA with the human genome reference sequence (GRCh37) using Bowtie2 [20]. Single nucleotide polymorphisms (SNPs) were called with Genome Analysis Toolkit (GATK, version 2.5) SNPs were filtered as following criteria. (i) SNP candidates were called from a sequencing region with a minimum coverage of 10×, with at least 5× the appearance of a minor allele. (ii) Among these candidate SNPs, only variants with an average quality score of 20 (Phred scale) were chosen. (iii) Allelic frequency (100 × Minor allele counts / Total allele counts) of less than 20% were considered as a sequencing error, 20–80% were considered as a heterozygote, and over 80% were considered as a homozygote [21, 22]. We annotated genetic variants using the software ANNOVAR [23] and called copy number variation using the software CoNIFER [24].

### Association test analysis

By setting the phenotype as the change in hair count after the 6-month treatment with dutasteride (0.5 mg), quantitative regression-based association tests between phenotype and genotype were performed for all SNPs, following which quantitative association tests using linear regression were performed for the data generated from all subjects with unadjusted and age adjusted models. Furthermore, we conducted quantitative association tests with good- and poor-response groups as cases and controls, respectively. The case/control association test was conducted twice using 2 different standards for classification: upper half vs. lower half and top quartile vs. bottom quartile response to dutasteride. To enhance confidence in our selected candidates, Fisher’s exact tests were performed on the groups as well. All of the association tests were conducted using PLINK [25]. Multiple test corrections were performed using Bonferroni correction, taking into account linkage disequilibrium (LD) blocks with a threshold of *R*^*2*^ >0.8 using pairwise tagging in HaploView [26].

### Gene-based association analysis

Gene-based association tests were conducted using VEGAS [27] 4 times on the statistically significant SNPs, as determined by the 4 prior association tests, with over 10^5^ simulations of each gene. To identify a hidden association, i.e. the cumulative effect among exonic SNPs, one exonic variant was selected from each gene as a tagged SNP. Each SNP with a positive effect was rated as “+1” and with a negative effect as “-1”. Allele frequencies of good- and poor-response groups were compared that of an Asian population.

### Multifactor dimension reduction modelling

**Fig 1:**
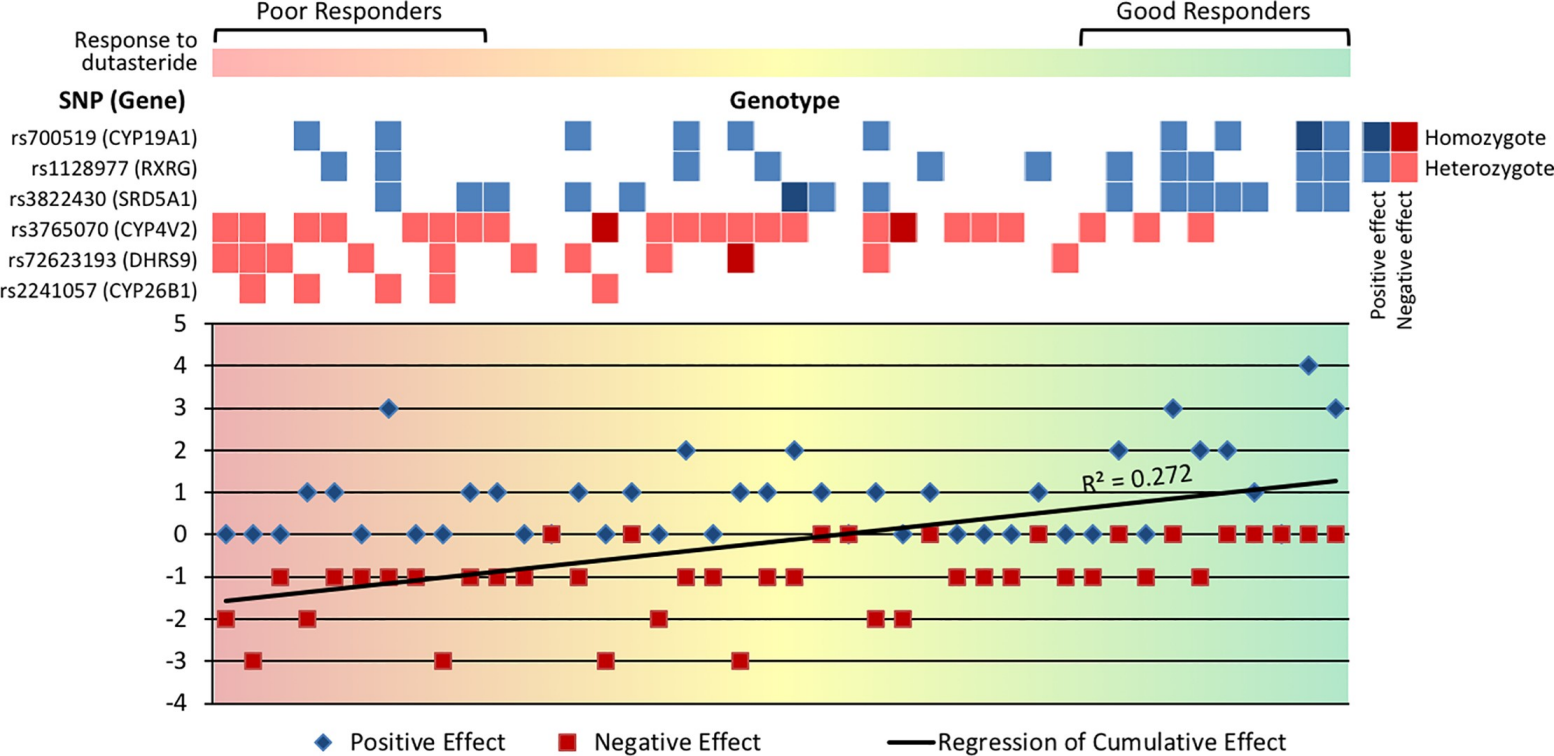
Genotypic landscape of 42 patients and the cumulative effect of each allele count and their positive or negative effect. In the upper figure, boxes represent the genotypes of each sample. Light-coloured boxes represent heterozygote SNPs and dark-coloured boxes represent homozygote SNPs. SNPs with negative effects and positive effects, as determined from the regression test, are in the red boxes and blue boxes, respectively. The lower figure shows cumulative effect by counting the number of SNPs. By considering SNPs with a negative effect as -1 and those with a positive effect as +1 as well as by applying regression tests to these cumulative scores, a positive relationship between the cumulative effect of SNPs and the change in hair count was observed. In both figures, samples are ordered along the X-axis from those showing the poorest to the best response in terms of change in hair count.

To evaluate the non-additive effect, Multifactor Dimension Reduction (MDR) [28], a statistical epistasis analysis method for genetic association, was conducted. We used the 6 exonic variation candidates in Fig 1 to perform a multifactor dimensionality reduction analysis using MDR 3.0.2 (https://www.multifactordimensionalityreduction.org). We classified the variations by their haplotypes and considered a poor responder group (class “1”) and a good responder group (class “2”) as discrete variables. Before the analysis, the attribute count range was configured as 1:6 to determine the number of attributes that fit the best model by considering the balancing accuracy and cross-validation consistency using all 6 SNPs.

## Results

In total, 42 out of 64 MPHL subjects enrolled in the previous phase III clinical trial that received dutasteride (0.5 mg) for 6 months agreed to participate and were included in this pharmacogenetic study (S2 Table). To evaluate non-genetic factors associated with response to dutasteride, we compared the age, height, and weight and the duration of MPHL between the 10 highest- and 10 lowest-ranked patients based on their hair count increase after 6 months of treatment; however, we did not find any significant differences among the subjects with respect to these parameters (S3 Table).

The outline of the genetic analysis is shown in S2 Fig. Subjects were sequenced with a customized targeted capture kit for next-generation sequencing. On average, 4.3 Gb were sequenced per sample, and 92.37% of the target region was covered over 30×, with a mean depth of coverage 296.3× (S4 Table). Among the 9524 SNPs called, 3430 were left in the target region after removing low quality, homogeneous SNPs, and singletons. From these SNPs, we could identify 86 exonic variants (34 non-synonymous, 49 synonymous, 1 stop-gain, and 2 unknown) and 1 splice-junction variant that affects protein coding. Copy number variation was assessed using CoNIFER [24]. We found 2 deletions in the poor-response group: 1 was found in *CYP4Z2P* of the poorest responder and the other in *CYP2A6* of 2 patients who showed 2^nd^ and 9^th^ poorest responses (S3 Fig). However, because of the small sample size, we could not determine the statistical significance of these deletions.

To identify variants associated with response to dutasteride, we first performed quantitative regression tests on all 42 samples’ genotypes and found 162 candidate variants that were significantly associated with an increase in hair count after dutasteride treatment (S5 Table). Interestingly, *ESR1* had 51 variants that were strongly associated with response to dutasteride, all of which are located in the intronic region.

Further, to identify the relevant genetic variants more precisely, we performed a case-control association test by comparing the good- and poor-response groups. We divided subjects into 2 groups based on the increase in hair count after treatment with dutasteride and set the upper half as cases and the lower half as controls. A total of 137 variants were significant with *p* <0.05, and *ESR1* was again determined to be the gene with the most number of significant variants (a total of 40) (S6 Table).

To identify genotypes associated with extreme phenotypes, we performed additional case-control association tests with the top and bottom quartiles of subjects and found 127 variants that were significant (S7 Table). Additionally, Fisher’s exact test on these 2 quartiles showed 53 variants that were more stringently associated with response to dutasteride (S8 Table). In addition to variants in *ESR1*, a few intronic variants in *DHRS9* were highly ranked in these 2 tests.

To identify the functional effects of SNPs, we summarized the exonic variants identified from these 4 association tests (Table 1). Synonymous SNP rs72623193 in the *DHRS9* gene was the most significant variant and the only exonic variant that passed all 4 association tests. A linear regression test revealed a negative effect of this SNP on response to dutasteride in (*β* = -11.70).

**Table 1:** SNPs in the exonic region that are statistically associated with response to dutasteride.

Chr	Position	SNP	Cyto	Allele frequency			Gene	Base	A. a.	Func. Impt.	*P*-value				
			band	Poor	ASN	Good		Change	Change		AQt	AQt*	H	Q	QF
2	169,952,166	rs72623193	2q31.1	0.25	0.13	0.00	*DHRS9*	T > C	-	GERP++ 4.17	**0.019**	**0.021**	**0.014**	**0.0098**	**0.047**
2	72,361,960	rs2241057	2p13.2	0.20	0.08	0.00	*CYP26B1*	A > G	L>S	Benign (0.78, 0.00)	**0.023**	**0.021**	**0.017**	**0.025**	0.106
										GERP++ 3.32					
1	165,389,129	rs1128977	1q23.3	0.10	0.11	0.25	*RXRG*	G > A	-	GERP++4.3	**0.034**	0.050	0.292	0.16	0.41
5	6,651,970	rs3822430	5p15.31	0.10	0.15	0.35	*SRD5A1*	A > G	-		**0.035**	**0.033**	0.082	**0.025**	0.13
5	6,652,009	rs8192186	5p15.31	0.10	0.15	0.35	*SRD5A1*	G > A	-		**0.035**	**0.033**	0.082	**0.025**	0.13
5	6,656,210	rs3736316	5p15.31	0.10	0.15	0.35	*SRD5A1*	G > A	-		**0.035**	**0.033**	0.082	**0.025**	0.13
15	51,507,968	rs700519	15q21.2	0.10	0.18	0.25	*CYP19A1*	G > A	R>C	Impt (0.04, 0.00)	**0.040**	**0.044**	0.072	0.212	0.408
										GERP++ 4.4					
4	187,122,355	rs3736456	4q35.2	0.35	0.37	0.15	*CYP4V2*	T > C	-		**0.041**	0.050	0.183	0.074	0.273

ASN, 1000G Phase1 East Asian; A. a, Amino acid; AQt, All Quantitative association test; Chr, Chromosome; Func Impt, Functional Important; Good, Good responders; H, Half case-control association test; Poor, Poor responders; Q, Quartile case-control association test; QF, Quartile Fisher’s exact test.

*Age adjusted

Listed SNPs are associated with good/poor response in the group-based tests or are predicted to be functionally important. Forma numbers were predicted with SIFT (0–0.05, where lower scores are more important) and PolyPhen2 (0–1, where higher scores are more important). GERP++ score indicates evolutionally conserved regions (GERP++>2). *P*-values < 0.05 are indicated in bold. None of these *p*-values survived multiple test correction due to the small sample size. Regardless, we identified the same candidate locus ranked from 4 different association tests and considered it worthy of reporting.

Two missense mutations, rs2241057 in *CYP26B1* and rs700519 in *CYP19A1*, were also associated with response to dutasteride. Rs2241057 showed a negative effect (*β* = -17.66) and was found only in subjects of the poor-response group with heterozygote genotypes. On the other hand, rs700519 had a positive effect (*β* = 10.56) with a tendency of increased allelic frequency in good responders. However, these mutations were not predicted to cause a dramatic damage in gene function. PolyPhen2 predicted both mutations as ‘benign,’ whereas SIFT predicted the latter mutation as deleterious.

To determine the gene-level association, we performed gene-based association tests using previously determined significant genotypes of the SNPs described above (Table 2). *DHRS9* and *ESR1* passed all 4 gene-based association tests. Interestingly, most of the variants in these genes had shared haplotypes (S4A and S4B Fig) and the same type of effect in linear regressions. SNPs in *DHRS9* had a negative effect (β ≤ -10.13) compared to SNPs in *ESR1* (β ≥ 8.378) (S9 Table). Although they did not pass all of the association tests, *RARG*, *H3F3A*, *SRD5A1*, and *HDAC1* are also suggested to have substantial effects on response to dutasteride. SNPs in *SRD5A1* showed a positive association with drug response (β ≥ 9.095). An LD plot of these SNPs shows that they are significantly associated (*p* < 0.05) in a 25-Kb long region of *SRD5A1* (S4(C) Fig).

**Table 2:** Results of the gene-level association tests.

Chr	Gene	Start	Stop	*P*-values			
				AQt	H	Q	QF
2	*DHRS9*	169,629,544	169,660,923	**0.007**	**0.018**	**0.005**	**0.009**
6	*ESR1*	152,053,323	152,466,101	**0.04**	**0.046**	**0.048**	**0.047**
12	*RARG*	51,890,619	51,912,303	**0.012**	0.318	**0.022**	**0.056**
1	*H3F3A*	224,317,043	224,326,326	**0.037**	0.594	0.094	0.166
5	*SRD5A1*	6,686,499	6,722,675	**0.04**	0.135	0.063	0.219
1	*HDAC1*	32,530,294	32,571,811	**0.029**	0.106	0.096	0.357

AQt, All Quantitative association test; H, Half case-control association test; Q, Quartile case-control association test; QF, Quartile Fisher’s exact test.

This table shows genes significantly associated with response to dutasteride (*p* <0.05). This test was performed using VEGAS, with start-stop positions in hg18.

By taking 1 exonic variant from each gene as a tagged SNP, each variant with a positive effect was treated as a protective factor and that with a negative effect was treated as a risk factor. We empirically calculated the cumulative effect by adding allele counts of each SNP and subtracting the risk factors from the protective factors. The regression of the cumulative effect was positively correlated with response to dutasteride (Fig 1). Allele frequencies of each SNP were different between poor and good responders. Asian frequency obtained from the 1000 Genomes Project was in-between allele frequencies of the poor-/good-response groups in all SNPs except for *CYP4V2* (S5 Fig).

**Fig 2:**
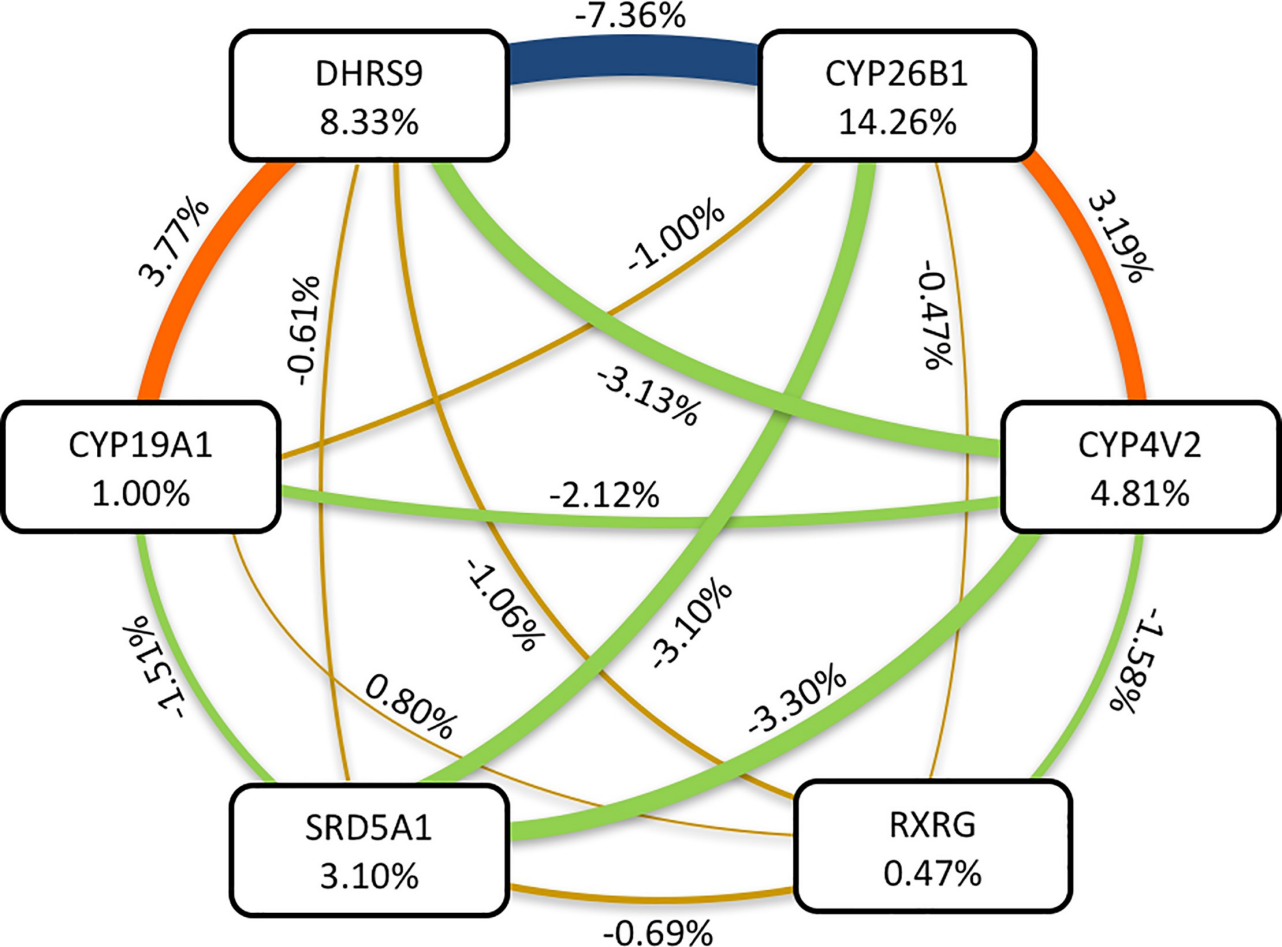
Entropy-based interaction graph for exonic variations drawn with Multifactor Dimension Reduction (MDR) analysis of good-/-poor responders treated as cases/controls. Positive entropy indicates information gain or synergistic effect (i.e. non-additive), whereas negative entropy indicates redundancy or lack of information gain. Model statistics as well as single and pairwise attribute values for information gain and interactions are listed in S10–S13 Tables.

To examine hidden associations, also called as gene-gene interactions or epistasis, we performed a non-parametric, genetic model-free data mining strategy MDR by using the 6 loci described above. The results of the cross-validation statistics used for training and testing the MDR model and the predicted class of each genotype combination were shown in S10 and S11 Tables. For a detailed model description of values in entropy-based measures for information gain (IG) and interaction (I), see S12 and S13 Tables. For equations used to calculate IG and I [28]. This model had cross-validation consistency of 10 with a balanced accuracy of 87.19% (Fig 2 and S10–S13 Tables). Polymorphisms in *CYP19A1* and *DHRS9* synergistically interact (i.e. non-additive or exclusive-OR interactions) and had the most entropy (3.77%), followed by *CYP26B1* and *CYP4V2* (3.19%). *DHRS9* and *CYP26B1* are highly additive (redundancy or lack of information gain, -7.35%). This result indicates existence of nonlinear interactions between SNPs that underlie the cumulative effect.

## Discussion

Dutasteride is a dual inhibitor of type I and II 5ARs and has proven to be an effective and tolerable treatment modality for MPHL. It improves hair growth in a dose-dependent manner by reduction of serum and scalp DHT [16]. The response to dutasteride varies in each individual, but genetic factors related to individual differences are unclear. In this study, we found several candidate regions associated with response to dutasteride in MPHL treatment through genotypic association tests.

In our study, the most significant exonic variant was rs72623193 located in *DHRS9*, that was ranked the highest in the gene-based tests. DHRS9 (dehydrogenase/reductase SDR family member 9) has been identified as a 3α-hydroxysteroid dehydrogenase (3α-HSD) [29]. It is a microsomal enzyme whereas other 3α-HSDs in the aldoketoreductase gene superfamily (AKR1C1–AKR1C4) are cytosolic enzymes. This enzyme is expressed in the human epidermis, hair follicles, and sebaceous glands [30, 31]. Since DHRS9 is involved in the synthesis of DHT from 3α-androstanediol [29], upregulation would facilitate the backdoor pathway to DHT in the scalp tissue even when 5AR is inhibited by dutasteride (S6(A) Fig). A previous study showed that there was negative correlation between the change in hair count and the percent change of scalp DHT after treatment with the 5AR inhibitor [16].

Additionally, DHRS9 possesses retinol dehydrogenase (RolDH) activity [32, 33], which converts retinol to retinal in retinoid acid (RA) synthesis (S6(B) Fig). Because DHRS9 can oxidize retinol that is bound to the cellular retinol-binding protein while many other RolDHs cannot [34, 35], it is regarded as a physiological enzyme [35]. Its expression changes during hair cycling in mice and increases in hair follicles of C57BL/6J mice that frequently develop dorsal skin alopecia. DHRS9 also increased in the hair follicles of patients with central centrifugal cicatricial alopecia [30]. Previous studies demonstrated that RA synthesis and the RA signalling system are related to hair growth and cycling [35, 36]. Furthermore, RA was reported to regulate bone morphogenetic protein and Wnt signalling pathways, which are involved in hair growth [37].

Rs2241057, the second most significant exonic SNP in this study, is located on *CYP26B1*, which is also involved in regulation of RA level. RA is metabolized by cytochrome P450 26 family members (CYP26A1, B1, and C1) (S6(B) Fig) [38]. CYP26A1 and CYP26B1 are expressed and are RA-inducible in both reconstructed and *in vivo* human epidermis [39, 40], but CYP26B1 was suggested to be more effective under physiological conditions [41], CYP26B1 is able to inactivate both all-*trans*-RA and 9-*cis*-RA [42]. Additionally, we found that rs1128977 on *RXRG* is associated with response to dutasteride. This gene encodes retinoid X receptor-γ (RXRG), a nuclear receptor that is activated by 9-*cis*-RA. Taken together, these findings strongly suggest that RA metabolism and the RA signalling pathway are associated with response to dutasteride in MPHL, although mechanism of action needs to be elucidated in future studies.

We also found that response to dutasteride may be associated with variants of genes involved in steroid hormone metabolism, such as *SRD5A1*, *ESR1*, and *CYP19A1* (S6(A) Fig). The product of *SRD5A1* is type I 5AR, which is a target of dutasteride. Dutasteride inhibits both type I and II 5ARs, while finasteride selectively inhibits type II 5AR. Therefore, a variation in *SRD5A1* might affect the subject’s response to dutasteride during treatment for MPHL. However, *SRD5A2* encoding type II 5AR was not associated with response to dutasteride.

In addition to androgens, it has long been known that oestrogen also effects hair follicle growth by binding locally expressed oestrogen receptors (ERs) [43]. *ESR1* encodes ERα, which is 1 of 2 distinct isoforms of ERs. *CYP19A1* converts androstenedione to estrone (E1) and testosterone to 17β-estradiol (E2). It has been reported that ERα is maximally expressed during telogen, and E2 inhibits hair growth [44, 45]. Moreover, E2 modifies androgen metabolism in pilosebaceous units [43].

In this study, no individual variant passed the filtering criteria after multiple tests correction was applied. This might be due to the small number of participants enrolled in this study. However, we cannot exclude the effects of interaction between multiple variants, considering that MPHL is very complex, and the hair-count change in each patient after treatment was distributed continuously and was not clearly divided into different groups. We hypothesize that the individual difference in response to dutasteride comes from the cumulative effects of multiple variants rather than that of a single responsible variant and depicts a genotypic landscape with good correlation between the allele frequency scores of the tagged exonic SNPs and response to dutasteride. Results from MDR analysis are also consistent with the existence of an interaction between SNPs. Therefore, we propose that these SNPs, when used simultaneously, could be suitable markers for clinicians to predict response to dutasteride in patients with MPHL.

In conclusion, we identified candidate variants associated with response to dutasteride for MPHL. According to the association tests, potential candidate variants are located on *DHRS9*, *CYP26B1*, *ESR1*, *SRD5A1*, *CYP19A1*, and *RXRG*. These genes encode proteins involved in the backdoor pathway of DHT synthesis, RA metabolism and the RA signalling pathway, and steroid hormone metabolism. To the best of our knowledge, this study is the first to show genetic variation associated with drug response in patients with MPHL. Moreover, this study could be developed further to investigate the cumulative effect of multiple variants. Our findings would contribute to the investigation on the pathophysiology of MPHL and can potentially help clinicians improve treatment of MPHL patients by using 5AR inhibitors.

## Supporting information

S1 Table List of the target genes. (XLSX)

S2 Table Epidemiologic data of the individual subjects. (XLSX)

S3 Table Epidemiological characteristics of the enrolled subjects with MPHL who had been treated with dutasteride (0.5 mg) for 6 months. (XLSX)

S4 Table Sequencing summary of 42 subjects. (XLSX)

S5 Table List of SNPs from quantitative linear regression test under p-value < 0.05. (XLSX)

S6 Table List of SNPs from upper half (n = 21) and lower half (n = 21) case-control association test under p-value < 0.05. (XLSX)

S7 Table List of SNPs from extreme phenotypes (n = 10 on each) case-control association test under p-value < 0.05. (XLSX)

S8 Table List of SNPs from extreme phenotypes (n = 10 on each) case-control Fisher's exact test under p-value <0.05. (XLSX)

S9 Table Genes and the number of SNPs commonly and significantly associated (p <0.05) with response to dutasteride as determined by 4 association tests. Effect, |β|, and R2 show the minimum to maximum range for each SNP used based on quantitative regression test results.
(XLSX)

S10 Table Results of the cross-validation statistics used for training and testing the MDR model with the 6 genotyped exonic SNPs. (XLSX)

S11 Table Predicted class of each genotype combination. Genotype combination is in order of SNPs in *DHRS9*, *CYP26B1*, *RXRG*, *SRD5A1*, *CYP19A1*, and *CYP4V2* used in the MDR model, and their number of alternate alleles noted as 0, 1, and 2.
(XLSX)

S12 Table Single attribute values in entropy-based measures for information gain and interaction depicted in Fig 2. (XLSX)

S13 Table Pairwise attribute values in entropy-based measures for information gain and interaction depicted in Fig 2. (XLSX)

S1 Fig Representative phototrichogram for the measurement of hair counts. Total hair counts and growth rate were measured on 1 cm^2^ circular area of clipped hair, selected from the anterior edge of the balding area on the vertex. A small cosmetic ink tattoo was marked in the center of the selected area to identify the same area of measurement at every measurement (yellow arrow). The phototrighogram was converted into dot map, and then converted to hair counts using a computer imaging system.
(PNG)

S2 Fig Schematic representation of SNP calling, variant filtering steps, and statistical analysis workflow. Tools used for each step are marked in blue; number of variants left after each filtering step are marked in red. After filtration and quality control, 4 different statistical analyses were applied at the SNP and gene levels. First, linear regression tests were applied to all samples; then, we divided our samples in to 2 groups for the case/control association test. This test has been applied twice by taking half of the samples as cases/controls and quartiles of the poor-/good-response groups. Last, Fisher’s exact tests have been applied to the poor-/good-response groups.
(TIF)

S3 Fig Copy number variations found in the poor-response group. **Red-marked samples represent poor responders and blue-marked samples represent good responders.** (A) Deletion in *CYP4Z2P* in the poorest responder patient (A037; hair count change of -22.5). This is a novel deletion that has not previously been reported [1]. (B) Deletion in *CYP2A6* in a patient with the 2^nd^ poorest response (A007; hair count change of -10.5) and another patient with the 9^th^ poorest response (A043; hair count change of -3.0). This deletion was reported to be commonly found in 20% of the Asian population (Lee C, Seo JS et al. Discovery of common Asian copy number variants using integrated high-resolution array CGH and massively parallel DNA sequencing. Nature Genetics, 2010; 42: 400–405)
(PDF)

S4 Fig Linkage disequilibrium (LD) blocks of variants in genes associated with drug response in MPHL treatment. Green-coloured rs numbers are SNPs in coding sequences, and black-coloured rs numbers are SNPs in intronic regions. (A)Variants in the LD blocks of *ESR1*. (B) Variants in the LD block of *DHRS9*. (C) Variants in the LD block of *SRD5A1*.
(PDF)

S5 Fig Comparison of the allele frequency of each group as determined by the present study with the Asian allele frequency obtained from the 1000 Genomes Project. Asian frequency was almost in-between allele frequencies of the poor-/good-responders. ASN, Asian population; good, good-responders; poor, poor-responders.
(PDF)

S6 Fig Overview of metabolic pathways of steroids and reitnoids. (A) Metabolic pathways of steroids. DHT is normally produced from testosterone in target tissues (conventional pathway, black box), but is also synthesized from androstanediol (back-door pathway, blue box) by a reaction in which DHRS9 is involved. CYP19 converts androstenedione into estrone and testosterone into estradiol (oestrogen biosynthesis, pink box). (B) Overview of retinoid metabolism in target cells. Retinol is transported into the cell where binds CRBP, which is oxidized to retinal by DHRS9. Retinal is further oxidized to retinoic acid. CYP26 is involved in retinoic acid metabolism.ALDH1, retinal dehydrogenase 1; CRABP2, cellular retinoic acid binding protein II; CRBP, cellular retinol-binding protein; CYP17, cytochrome P450 17 family members; CYP19, cytochrome P450 19 family members; CYP26, cytochrome P450 26 family members; DHRS9, dehydrogenase reductase member 9; DHT, dihydrotestosterone; 3*α*-HSD, 3*α*-hydroxysteroid dehydrogenase; 17*β*-HSD, 17*β*-hydroxysteroid dehydrogenase.
(PDF)
