# Supplementary figures and images for: Genetic variations associated with response to dutasteride in the treatment of male subjects with androgenetic alopecia
Source: PLoS One. 2019 Sep 16;14(9):e0222533. doi: 10.1371/journal.pone.0222533 (PMC6746394; doi:10.1371/journal.pone.0222533)

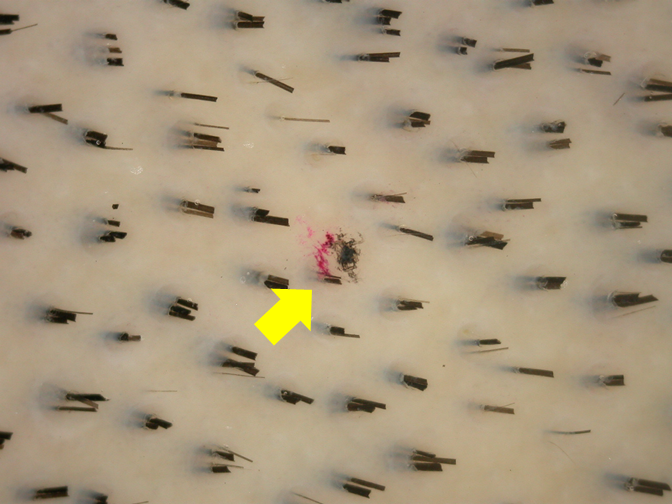

Supplement: S1 Fig — Total hair counts and growth rate were measured on 1 cm2 circular area of clipped hair, selected from the anterior edge of the balding area on the vertex. A small cosmetic ink tattoo was marked in the center of the selected area to identify the same area of measurement at every measurement (yellow arrow). The phototrighogram was converted into dot map, and then converted to hair counts using a computer imaging system. (PNG) [file pone.0222533.s014.png]

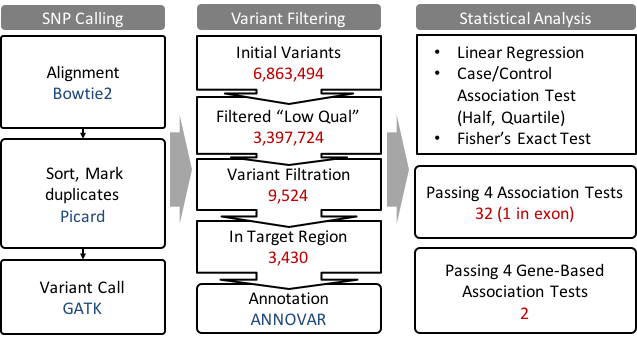

Supplement: S2 Fig — Tools used for each step are marked in blue; number of variants left after each filtering step are marked in red. After filtration and quality control, 4 different statistical analyses were applied at the SNP and gene levels. First, linear regression tests were applied to all samples; then, we divided our samples in to 2 groups for the case/control association test. This test has been applied twice by taking half of the samples as cases/controls and quartiles of the poor-/good-response groups. Last, Fisher’s exact tests have been applied to the poor-/good-response groups. (TIF) [file pone.0222533.s015.tif]

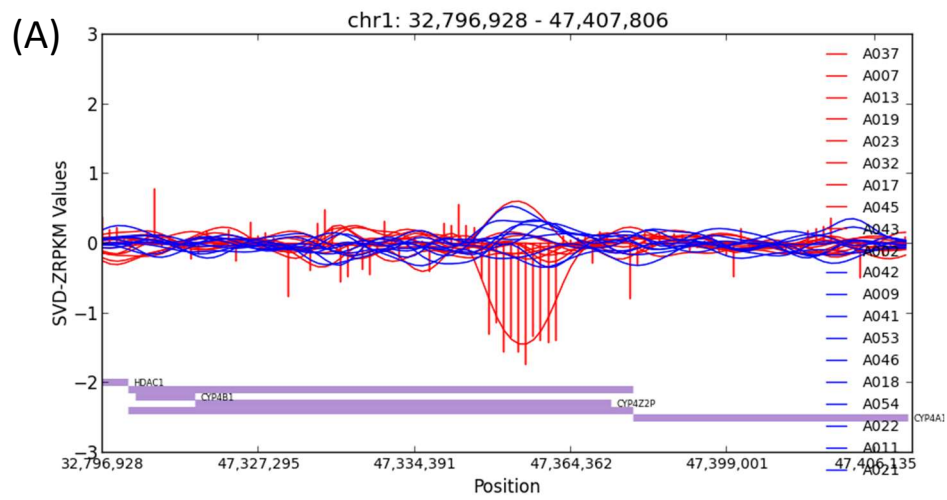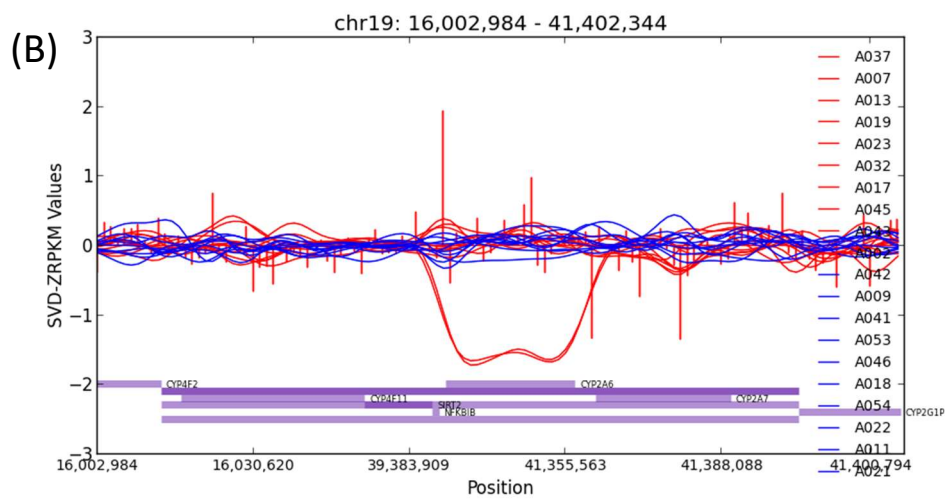

Supplement: S3 Fig — Red-marked samples represent poor responders and blue-marked samples represent good responders. (A) Deletion in CYP4Z2P in the poorest responder patient (A037; hair count change of -22.5). This is a novel deletion that has not previously been reported [1]. (B) Deletion in CYP2A6 in a patient with the 2nd poorest response (A007; hair count change of -10.5) and another patient with the 9th poorest response (A043; hair count change of -3.0). This deletion was reported to be commonly found in 20% of the Asian population (Lee C, Seo JS et al. Discovery of common Asian copy number variants using integrated high-resolution array CGH and massively parallel DNA sequencing. Nature Genetics, 2010; 42: 400–405) (PDF) [file pone.0222533.s016.pdf]

(A)

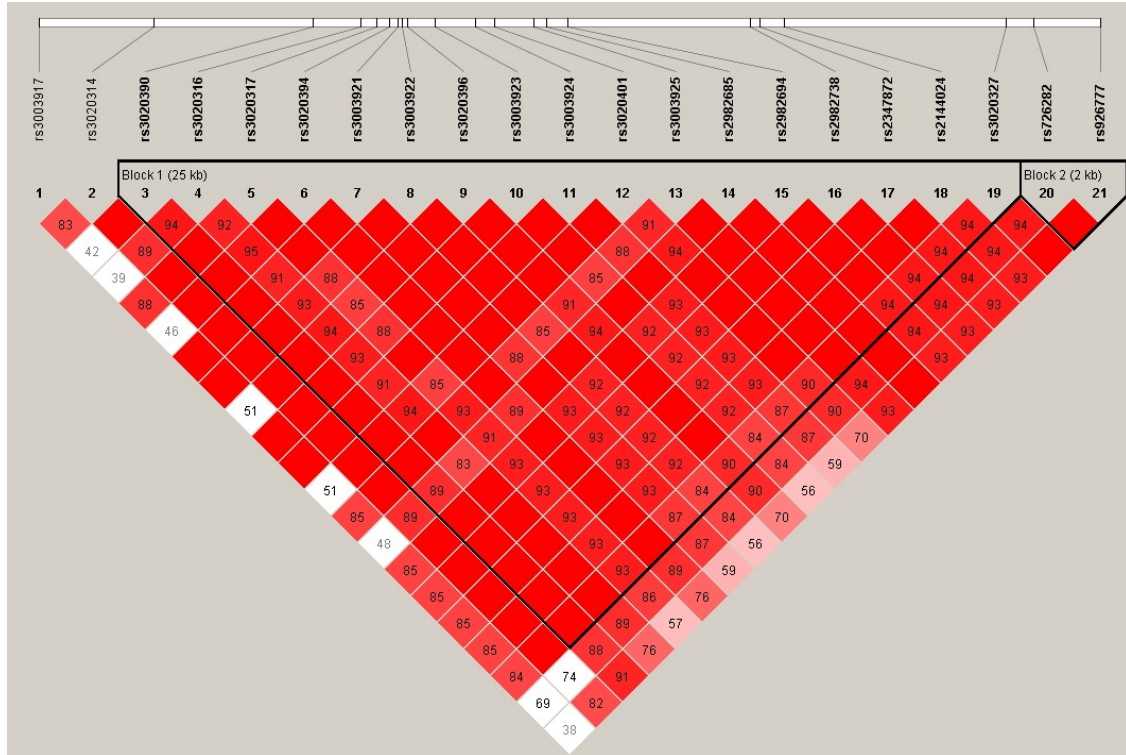

(B)

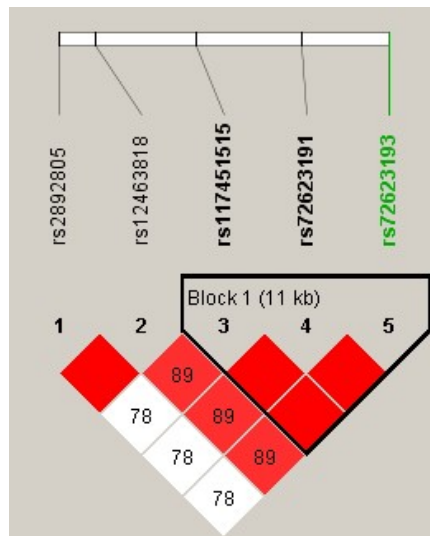

(C)

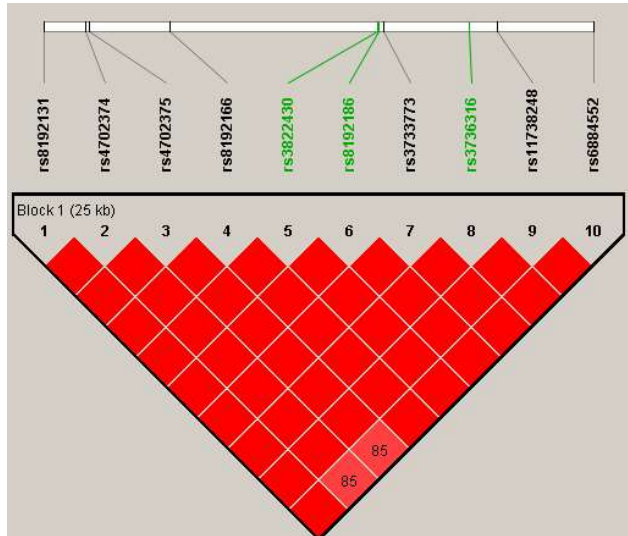

Supplement: S4 Fig — Green-coloured rs numbers are SNPs in coding sequences, and black-coloured rs numbers are SNPs in intronic regions. (A)Variants in the LD blocks of ESR1. (B) Variants in the LD block of DHRS9. (C) Variants in the LD block of SRD5A1. (PDF) [file pone.0222533.s017.pdf]

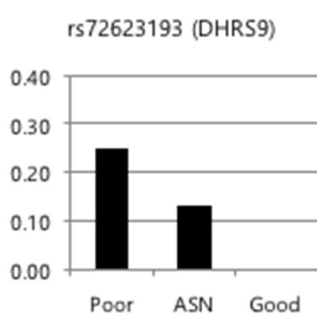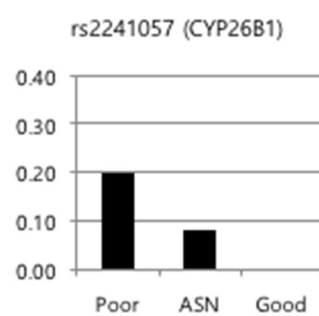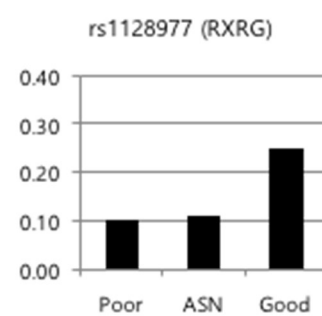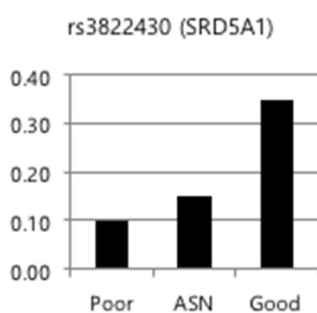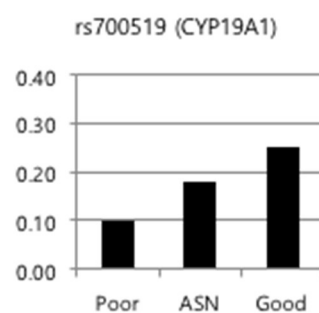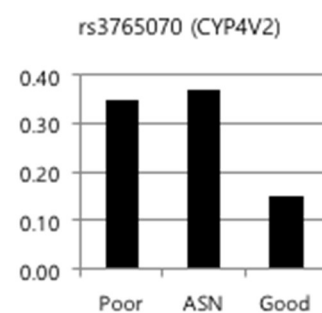

Supplement: S5 Fig — Asian frequency was almost in-between allele frequencies of the poor-/good-responders. ASN, Asian population; good, good-responders; poor, poor-responders. (PDF) [file pone.0222533.s018.pdf]

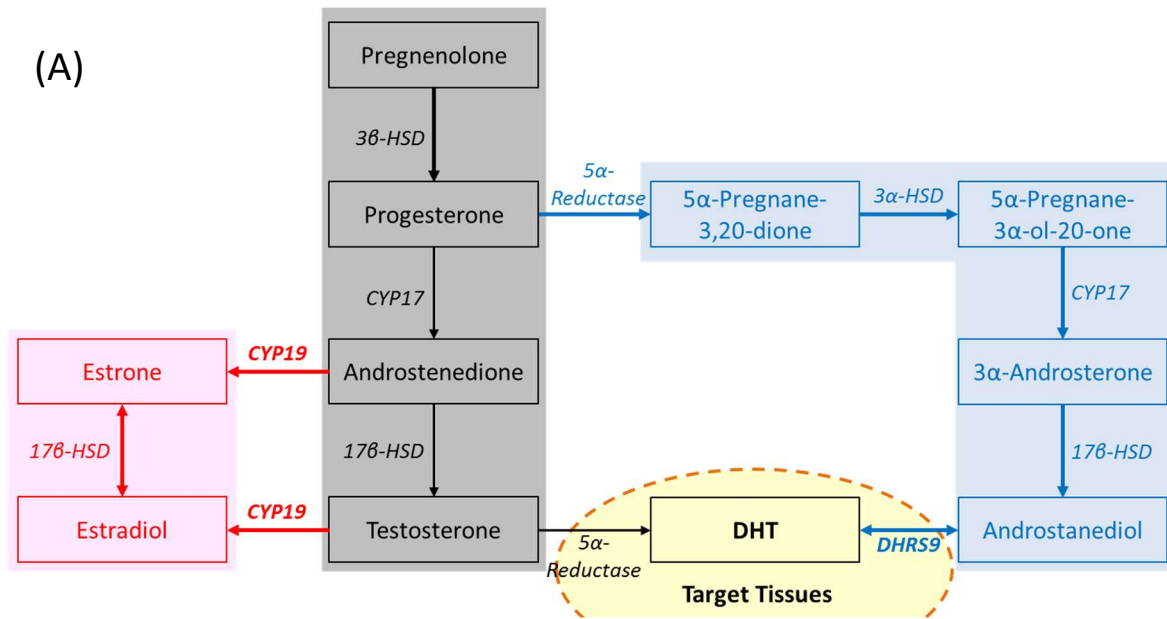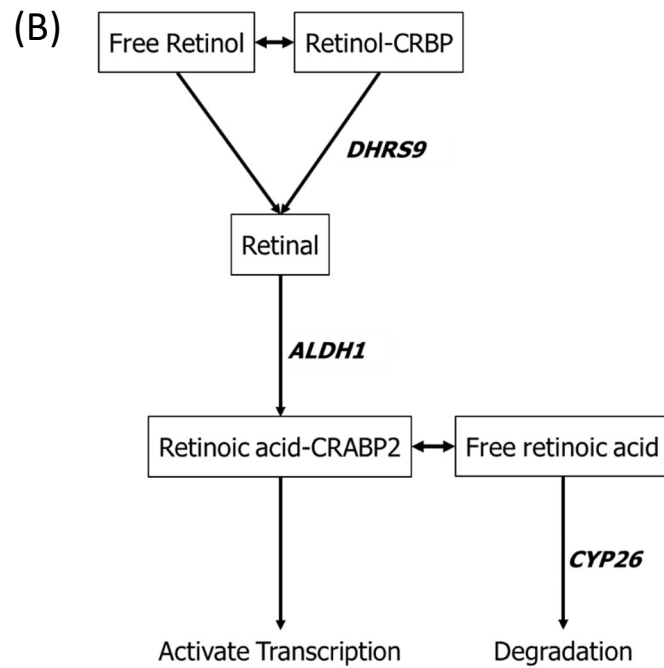

Supplement: S6 Fig — (A) Metabolic pathways of steroids. DHT is normally produced from testosterone in target tissues (conventional pathway, black box), but is also synthesized from androstanediol (back-door pathway, blue box) by a reaction in which DHRS9 is involved. CYP19 converts androstenedione into estrone and testosterone into estradiol (oestrogen biosynthesis, pink box). (B) Overview of retinoid metabolism in target cells. Retinol is transported into the cell where binds CRBP, which is oxidized to retinal by DHRS9. Retinal is further oxidized to retinoic acid. CYP26 is involved in retinoic acid metabolism.ALDH1, retinal dehydrogenase 1; CRABP2, cellular retinoic acid binding protein II; CRBP, cellular retinol-binding protein; CYP17, cytochrome P450 17 family members; CYP19, cytochrome P450 19 family members; CYP26, cytochrome P450 26 family members; DHRS9, dehydrogenase reductase member 9; DHT, dihydrotestosterone; 3α-HSD, 3α-hydroxysteroid dehydrogenase; 17β-HSD, 17β-hydroxysteroid dehydrogenase. (PDF) [file pone.0222533.s019.pdf]
